# Analysis of Reverse Phase Protein Array Data: From Experimental Design towards Targeted Biomarker Discovery

**DOI:** 10.3390/microarrays4040520

**Published:** 2015-11-03

**Authors:** Astrid Wachter, Stephan Bernhardt, Tim Beissbarth, Ulrike Korf

**Affiliations:** 1 Statistical Bioinformatics, Department of Medical Statistics, University Medical Center Goettingen, Humboldtallee 32, D-37073 Goettingen, Germany; E-Mails: astrid.wachter@med.uni-goettingen.de (A.W.); tim.beissbarth@ams.med.uni-goettingen.de (T.B.); 2 German Cancer Research Center (DKFZ); E-Mail: s.bernhardt@dkfz-heidelberg.de

**Keywords:** cancer, reverse phase protein array, reverse phase protein arrays (RPPA), signaling pathways, immunoassay, antibody

## Abstract

Mastering the systematic analysis of tumor tissues on a large scale has long been a technical challenge for proteomics. In 2001, reverse phase protein arrays (RPPA) were added to the repertoire of existing immunoassays, which, for the first time, allowed a profiling of minute amounts of tumor lysates even after microdissection. A characteristic feature of RPPA is its outstanding sample capacity permitting the analysis of thousands of samples in parallel as a routine task. Until today, the RPPA approach has matured to a robust and highly sensitive high-throughput platform, which is ideally suited for biomarker discovery. Concomitant with technical advancements, new bioinformatic tools were developed for data normalization and data analysis as outlined in detail in this review. Furthermore, biomarker signatures obtained by different RPPA screens were compared with another or with that obtained by other proteomic formats, if possible. Options for overcoming the downside of RPPA, which is the need to steadily validate new antibody batches, will be discussed. Finally, a debate on using RPPA to advance personalized medicine will conclude this article.

## 1. Analytical Needs of Personalized Oncology

With the development of personalized therapeutics for oncology, the systematic and targeted analysis of selected proteins in tumor tissues is currently receiving increasing interest. As a matter of fact, the majority of proteins that can be by targeted by therapeutics are cell surface receptors or proteins involved in cellular signaling. Yet, the number of clinically approved biomarkers is much lower than the number of proteins that could potentially be inhibited with small molecule drugs and therapeutic antibodies. Apart from prime examples such as therapeutic antibodies targeting the HER2 receptor in breast cancer or CD20 in malignant lymphoma, the majority of cancer types are still in need of suitable biomarkers that allow patient-tailored treatment. Hence, a systematic characterization of those membrane receptors and signaling proteins that can be targeted with the repertoire of currently available targeted drugs would be required in the first place to advance the personalized treatment of cancer patients. Therefore, immunoassay-based technologies present a promising experimental approach since they can quickly deliver quantitative information on the expression of already known target proteins.

Screening clinical tissues for target proteins of potential pharmaceutical interest requires large numbers of well-documented clinical samples to yield statistically relevant data. High capacity platforms such as reverse phase protein arrays (RPPA), for example, are therefore well suited for this purpose. However, targeted proteomics requires first of all profound insights into cellular processes underlying cellular transformation and metastasis and a good knowledge of biochemistry. Fundamental changes of the cellular proteome occur immediately post-excision, a process described as cold ischemia. In fact, clinical tissues are still alive and biochemical processes will still proceed as long as enzymes are not inactivated by freezing or other suitable measures [1]. Enzymatic activities can also be re-activated during sample-thawing and lysate preparation. With this in mind, suitable measures are required to preserve the cellular proteome during all working steps of sample preparation and sample handling. Especially, the phosphoproteome is subjected to fast regulation since the turnover rates of kinases and phosphatases are high, and protein kinases will particularly benefit from the ample ATP reservoir of cancer cells. Several studies aimed for quantifying the turnover of protein composition as well as the proteome during cold ischemia. Apparently, 30% of all proteins change within the first half hour after surgical excision [2]. However, after 30 min of cold ischemia, about 75% of protein and phosphoproteins seem to be stable until 24 h after surgery [3]. Therefore, biochemical processes occurring post-excision need to be taken into account as an important pre-analytical factor that will influence the resulting sample quality and require standardized procedures regarding tissue handling as well as sample preparation to ensure comparable sample quality. Hence, to set-up a bio-bank, clinical tissues need to be processed under consistent conditions over many years to exclude artifacts resulting from tissue handling and storage.

## 2. Use of RPPA for Biomarker Discovery

The RPPA principle relies on the very simple dot-blot idea of probing for specific proteins in crude lysates that were printed as small dots on a solid phase carrier. This simple concept was adapted to robotics-based arraying to achieve a higher accuracy to fulfill the requirements of a quantitative analysis [4]. Tumor lysates obtained by microdissection were profiled for the phosphorylation state of cancer-relevant signaling proteins by printing each sample as serial dilution onto nitrocellulose coated glass slides. This proof-of-principle approach illustrated that AKT signaling is enhanced at the tumor invasion front. Technically, this example documented also that immunoassays can be carried out in multiplex by using only a single antibody for each target protein [4]. In the following years, this straightforward idea was adapted to a highly versatile immunoassay platform, mostly by exploiting technical advancements made in robotics, signal detection and by the introduction of elaborated bioinformatics approaches that serve the purpose of data normalization and data analysis.

**Figure 1:**
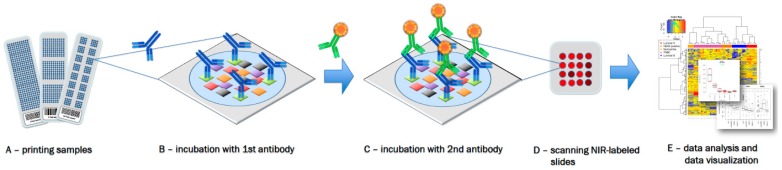
Reverse phase protein arrays (RPPA) experimentation involves (**A**) printing of samples in a neatly organized array format onto, for example, nitrocellulose-coated glass slides; (**B**) Incubation with a highly, target-specific primary antibody to detect proteins-of-interest, or a certain phosphorylation sites; (**C**) Signal detection of the primary antibody is commonly performed by fluorescence, chemiluminescence or colorimetric methods; (**D**) Target intensities are quantified after scanning and analyzing signal intensities of individual spots; (**E**) Data processing and quality control can be performed with the R-package RPPanalyzer (Table 1), for example.

Today, up to a few thousand individual samples can be analyzed in parallel by RPPA which illustrates the utility of this approach for the analysis of signaling networks or for screening of e.g., drugs [5] or microRNAs [6]. However, RPPA must not necessarily involve thousands of samples as experimentation can also be carried out at a small-scale format. Printing of replicate arrays—for example, printing 2, 3, 4 or 16 identical arrays on a single slide—is technically feasible with state-of-the-art arrayers and guarantees optimal usage of quite costly nitrocellulose-coated slides. Each subarray can be probed with a different primary antibody employing incubation chambers tailored towards the use of standard glass slides. That again illustrates the highly versatile character of RPPA (Figure 1).

Once samples of interest have been arrayed on the desired number of replicate slides in the format of choice, individual target proteins are detected with highly specific primary antibodies. As outlined previously, antibody validation presents a fundamental prerequisite. Thus, antibody cross-reactivity must be kept at a minimum for RPPA [7,8]. To verify antibody specificity, it is also possible to calculate a correlation factor between Western blot signals and array-based signals for a particular set of samples. However, it must be kept in mind that many proteins are expressed at similar levels in all cell lines and, in this instance, a correlation factor is of little use to judge antibody quality. Hence, even excellent and highly specific antibodies evaluated this way will yield mediocre correlation factors for the given set of samples. Visualization of primary antibodies captured by their corresponding target proteins on the array is carried out using suitable secondary antibodies, similar to Western blot or immunohistochemistry (IHC). Depending on high-resolution imaging systems available in the laboratory, secondary antibodies can be conjugated with different labels. Suitable are fluorescent dyes, for example, and preferentially those carrying near infrared (NIR) fluorescent dyes since most nitrocellulose surfaces still show substantial auto fluorescence in the visible range [9,10]. Likewise, enzyme-coupled secondary antibodies permit readout via chemiluminescence [11,12] or dye precipitation approaches, known from IHC [13,14].

In recent years, the highly robust character of NIR fluorescence-based detection has gained increasing attention in the field of RPPA. Compelling features of NIR fluorescence are low background on nitrocellulose-coated surface and with most biological materials so that a highly sensitive detection of target proteins is possible over several orders of magnitude. A restricted sensitivity in NIR detection was observed when working with highly vascularized clinical materials. However, in this instance, this obstacle can be overcome by macro- or microdissection. As an additional advantage, and in particular, outcompeting dye precipitation approaches, slides detected with NIR fluorescence show a very smooth background devoid of smears or other staining artifacts. For this reason, signal analysis does not require a spatial correction, which simplifies the data analysis procedure. In addition, NIR detection is technically highly robust and compatible with many different blocking buffer components and comprises only a single working step. As the only caveat, quite costly high-resolution NIR-laser based scanning systems are required for signal visualization. As a low-cost alternative, RPPA slides can also be read using a black/white scanning system. In this instance, signal detection must be carried out using dye precipitation kits [14,15]. Chemiluminescence signals can be monitored using standard X-ray film or CCD-camera based scanning systems, which are available as standard equipment in most laboratories. A downside of chemiluminescence is the requirement of accurate planning of slide exposure times since the emitted light fades away within a relatively short time, a process that also lowers the detection limit.

A few words should be said on handling slides during the detection process. A major demand is on avoiding drying of the relatively small nitrocellulose surface area while simultaneously aiming at a thorough removal of liquid remnants frequently restrained in the corners of the small incubation chamber. However, in routine settings, inter-array CV (coefficient of variation) values in a range <5% can be obtained, even for a manual slide detection approach [15]. In case staining artifacts such as smears or scratches are observed, slides should be excluded from the downstream data analysis procedure and detection should be repeated with a new slide. Freshly printed slides can be stored by freezing (−20 °C) and little deterioration in terms of signal quality is observed during the first two years of storage. Thus, printing a large number of slides is recommended for large projects since RPPA slides can be used as resource to preserve clinical samples and to validate a new working hypothesis at later time points in this way. Most RPPA groups control the quality of the printing process, especially when printing large slide numbers comprising tens or hundreds of identical slides, by staining few additional slides, e.g., every 10th slide of a printing run, with a total protein stain such as colloidal gold, Sypro Ruby or FAST Green FCF [15].

**Table 1:** Non-commercial software tools for RPPA data processing/analysis.

Tool	Implemented Quantification Methods	Implemented Normalization Methods	Accessibility	Comments	References
Supercurve	3 parameter logistic “SuperCurve” model, non-parametric model of Hu *et al.* [16]	variable slope normalization of Neeley *et al.* [17], surface adjustment using positive control spots of Neeley *et al.* [18]	[19], OOMPA R repository	classifier for quality control of RPPA of Ju *et al.* [20]	-
Normacurve	“SuperCurve” extension model: Non-parametric model of Hu *et al.* [16] with additive extension of sample and spatial effects		R package [21]	no package documentation available	[22]
Rppanalyzer	linear model, serial dilution curve of “Zhang *et al.* [23]	normalization with total protein dye, housekeeping protein normalization, median normalization, protein quantification assays	R package (CRAN, R-Forge)	wrapper function to “SuperCurve” model available	[24,25]
Rppapipe	-	-	web-based platform, R package (Bioconductor)	tool for analysis of pre-quantified and normalized datasets	[26]
Reverse Phase Protein Microarray Analysis Suite	-	normalization by a single normalizer or the geometric mean of several selected normalizers	VBA Excel macro	registration necessary	[27]
Miracle	3 parameter logistic “SuperCurve” model, logistic model of Tabus *et al.* [28], serial dilution curve of Zhang *et al.* [23] non-parametric model of Hu *et al.* [16]	median loading, variable slope normalization of Neeley *et al.* [17], housekeeping protein normalization	web application, R package “Rmiracle” (GitHub)	significance of relative sample differences by Dunnett’s test [29]	[30]

After signal detection has been completed, numeric values have to be assigned to all spots on all slides. This can easily be achieved by relying on standard scanner software or software tailored towards the analysis of array images. Most software approaches developed for RPPA data normalization and data analysis incorporate also additional experimental information and handle easily the integration of sample-specific information, e.g., clinical data or time points, as well as information on inhibitors in case RPPA is used for the analysis of biological experimentation.

## 3. Points to Consider before Sample Printing

### 3.1. Experimental Design

Successful biomarker discovery requires a careful reflection of all aspects that might play a role during data analysis. Key points comprise the experimental design that was chosen to approach the clinical question at hand. Thus, a definition of quality control (QC) measures and control samples meaningful for data analysis is required to analyze RPPA data. Using the same types of controls is also necessary to compare RPPA data across different RPPA platforms.

As RPPA enables a relative quantification of proteins in large sample sets, experimental effects that might take influence on raw data must be taken into account such as the dynamic range of measurements or spatial effects that might result from staining artifacts of the signal detection approach. In addition, sample loading has to match the signal detection range. To compensate experimental noise, for example, normalization approaches were developed which require additionally printed spots, such as sample dilution series or so-called loading control spots to account for uneven staining. All normalization methods require an array design that comprises printing of control samples and technical replicates to gain statistically relevant results. Apart from statistical aspects, the information inherent to replicate spots serves several other functions that become important during data analysis: on the one hand, it presents the basis to apply certain normalization methods, on the other hand, it also facilitates data comparability with already existing RPPA data sets, provided that the same controls were used.

For RPPA, two fundamentally different approaches exist. The first one is based on printing each sample as serial dilution, thus simultaneously providing a sufficient number of data points for downstream data analysis. Samples can also be printed in a single concentration which may be the approach of choice when the sample volume is too limited to allow for the preparation of serial dilutions, for example when working with scarce patient material. In this instance, it is highly important to choose a method for signal detection with a low experimental noise since this negatively impacts on data quality. However, co-printing serial dilutions of meaningful controls is also required in case samples are supposed to be printed in a single concentration to calibrate the signals obtained by the actual samples as realized in the second approach for RPPA. In this instance, samples are printed as three technical replicate spots to balance statistical power and to guarantee optimal spatial usage [22,28]. Relative protein quantification approaches, as commonly employed in RPPA, can also benefit from not simply aggregating sample replicates but using the information of individual replicate spots as measure of within-sample variability, as realized by the non-parametric estimation of protein expression levels by Li and coworkers as the Reno-approach [31].

### 3.2. Protein Quantification

**Figure 2:**
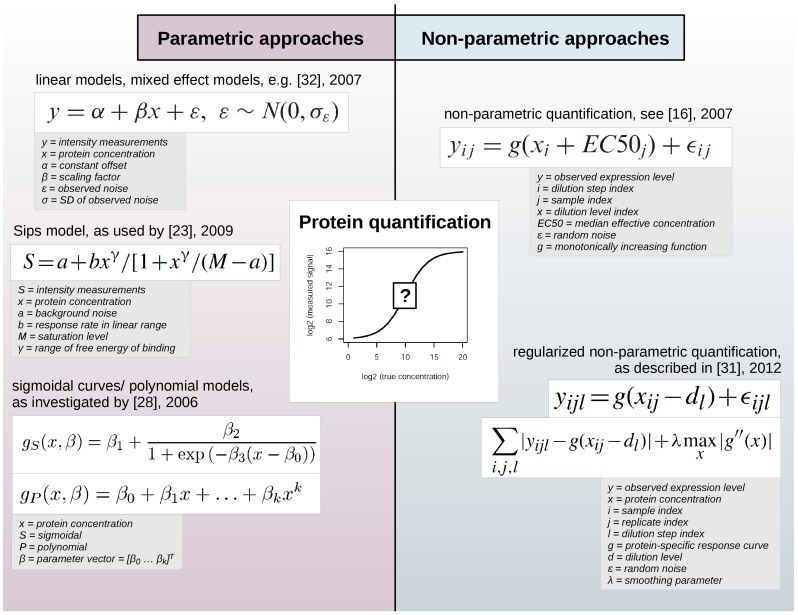
Parametric and non-parametric approaches for protein quantification in RPPA data sets. While parametric approaches employ pre-defined functions to describe the relationship between measured expression levels and protein concentration, non-parametric approaches are comparably more data-adaptive and use e.g., protein-specific response curves.

Two principally different mathematical approaches are employed to infer numeric values that reflect the expression level of proteins assessed by RPPA: parametric and non-parametric models (Figure 2).

Such statistical models contain so-called parameters that can either describe certain experimental characteristics, e.g., background noise or saturation level, and are defined before fitting the model to the data or are not defined initially. Thus, parametric models employ certain assumptions regarding the function used to describe the relation between the observed expression levels and the unobserved and yet unknown protein concentration. On the contrary, non-parametric models do not imply such a predefined form of the model, so that they are highly data-adaptive.

Parametric approaches have employed linear models [11,32] or sigmoidal curves [12,28] to determine a response curve, the relationship between the observed signal and the protein concentration. Zhang *et al.* [23] used the Sips model, which is similar to a logistic model, to determine the relationship between signals in successive dilution steps. Zhang and colleagues argued that the response curve depends on factors such as the target protein concentration and exposure time during signal detection, specific or non-specific interactions of reporter molecules with proteins of a particular sample or of the slide matrix. While the impact of experimental factors might be difficult to quantify and to incorporate into a statistical model, the serial dilution curve method allows the determination of experimentally meaningful model parameters that can be optimized further.

However, non-parametric methods seem to prevail due to their flexibility and their robust results. Non-parametric examples include the model of Hu *et al.* [16] which was based on the assumption that protein expression equals a non-parametric monotonically increasing function. The regularized approach by Li *et al.* [31] proposed an estimation of protein levels based on individual non-aggregated dilution series replicates to account for within-sample or within-group variability. Non-parametric parameters are more difficult to interpret but do not impose prior assumptions on experimental factors such as the reaction kinetics of the RPPA signal detection procedure.

### 3.3. Loading Control Normalization

Loading controls are required to account for measurable effects caused by properties of the starting material e.g., unequal total protein concentration or different cell numbers. Thus, a normalization step is required to permit the actual data analysis. Different approaches have been described which are based on adjusting the total protein concentration of individual samples to a pre-defined value prior to spotting. Protein quantification assays such as BCA or Bradford are frequently employed to determine the total protein concentration prior to spotting. In general, the accuracy of total protein assays is restricted by chemical inference with certain compounds and limited by a short linear range, not to mention the additional time needed for the experimental protocol. Alternatively, signals can be adjusted post detection. Post-printing normalization with a total protein dye requires additional slides of a print run to be stained with a total protein dye, for example Fast Green FCF, Sypro Ruby or colloidal gold. Antibody-detected slides are normalized based on data of a corresponding normalizer slide via a spot-specific correction factor that reflects the deviation of the protein concentration determined from the median of all spots. Target protein signals are then corrected via division by the correction factors, rescaling can be carried out by multiplication of spot intensities with the median of the corresponding normalizer array. Housekeeping proteins such as β-Actin have been used to normalize RPPA data [33,34]. However, even housekeeping proteins are subjected to biological regulation and have therefore limited these approaches.

Different normalization approaches were specifically tailored towards the needs of RPPA data analysis, e.g., median loading, loading control, variable slope, and invariable protein set normalization, as reviewed in [35]. Median Loading (ML) normalization considers row and sample effects as additive at the log scale. The sample effect is estimated from the median protein expression estimates of the samples across all arrays. The main assumptions of the median loading approach is that all arrays are printed in a consistent manner and that changes observed for up- or downregulated proteins can still be seen after median normalization [36]. A key idea behind this approach is that the majority of target proteins assessed by RPPA will be comparable for the majority of samples. However, if a low number of target proteins are probed by RPPA or only proteins subjected to regulation will be measured, the ML approach will be biased. Loading control incorporates similar ideas, yet the value reflecting median expression is calculated individually for each target protein and then subtracted from a particular sample [35].

Variable Slope (VS) normalization [17] takes into account the independent nature of individually stained RPPA slides. A slide-specific value is determined and included in the additive sample and row effect model in a multiplicative manner, thus yielding slightly different response curves for different slides. This approach was coupled with the “joint sample” model implemented in the suite of R packages “SuperCurve” (Table 1). These “joint sample” models use all the information of the array together with the individual protein concentrations for each sample to estimate parameters. The array information is based on assumptions such as that the surface chemistry and therefore the interactions of antibodies probed on a slide probed with a specific antibody are similar. For example, information available for each dilution point about rate of signal increase is used to yield improved estimates of protein concentration with a lower variance. “SuperCurve” relies on a three-parameter logistic equation to model the dependency of signal intensities from unknown protein expression values.

Recently, Liu *et al.* [35] employed an approach initially introduced for the analysis of high-throughput expression profiling data for loading control and variance stabilization, which was based on the invariant marker set concept [36]. This concept was adjusted to RPPA specific settings by introduction of a set of invariant proteins, so-called markers that form a virtual reference sample to normalize all samples. First, target protein signals are ranked and the variance is calculated across all samples, and data showing the highest rank variance are removed from the RPPA data set. This selection process is repeated until the number of target-protein derived data has reached a pre-determined number. Then, in this way, the reduced data set is trimmed further by removing the 25% highest and 25% lowest values. Next, averaging the remaining values of every protein across all samples generates the virtual reference sample (VS). The actual sample data is then normalized with respect to the virtual reference sample by lowess smoothing using an MA-plot approach as described in Pelz *et al.* [36]. So-called MA-plots or Bland-Altman-plots are often used to visualize the distribution of pairwise comparisons in transcriptomic experiments. The *x*-axis presents the log2 gene expression level and the *y*-axis reflects log2 fold-change with respect to a reference sample. This concept was employed by the VS approach and showed promising results with respect to loading effect correction and variance stabilization, and resulted in RPPA data that showed a good correlation with IHC/fluorescence *in situ* hybridization (FISH) data available for the same set of samples.

### 3.4. Spatial Normalization Methods

RPPA data quality depends strongly on the image quality obtained by the signal detection approach of choice. Certain detection methods, especially those comprising several working steps, can result in unevenly stained images caused by rim effects, for example. This spatial bias needs to be addressed by proper data analysis measures. The most obvious and most simple approach to tackle artifacts resulting from uneven staining or surface inhomogeneity is choosing a random sample distribution. However, in recent years, sophisticated methods were developed to improve RPPA data quality by co-printing of control spots.

In 2009, Anderson *et al.* [37] suggested to increase the statistical power by reducing the coefficient of variation so that variability resulting from spatial heterogeneity can be kept under control. This approach, termed “Array Microenvironment Normalization”, foresees printing a layout composed of an alternating checkerboard pattern of positive control spots and experimental sample spots. Controls were designed to match samples with respect to their total protein concentration as well as having a target protein concentration within the linear range of detection. Assuming that the relation of these concentrations is equal for all controls and independent from the position on the array, variations between individual control spots were attributed to spatial heterogeneity. Although the method improved the reproducibility of protein quantification, this approach is associated with a considerable increase of costs and efforts, as the number of samples that can be analyzed by RPPA is reduced.

The surface adjustment method developed by Neeley *et al.* [18] in 2012 requires a significantly lower number of control spots and relies on duplicate sample delivery in two differently defined printing patterns. This approach uses a generalized additive model to estimate a smoothed surface from which the positive control values are estimated for each spot of the array in relation to all other positive control spots. In case positive control spots were printed as dilution series, step-to-step differences can be used to perform an intensity-based adjustment by scaling each spot to the signal intensity of its immediate surface environment. With this method, a higher inter-slide reproducibility was obtained. However, the power of this approach was not directly compared with the one developed by Anderson and colleagues.

A recent approach from Kaushik *et al.* [38] accounts for spatial variability by using a simple bi-linear interpolation technique that yields a theoretical surface representing the spatial variation as basis for a calculation of correction factors. Inter-slide and intra-slide technical replicate agreement and intra-slide biological replicate agreement were determined in a 238-slide melanoma cell line study to evaluate this method. Intra-slide reproducibility of technical replicates was good and correlation between inter-slide replicates was high, however, the evaluation via correlation was not a good measure of data quality after normalization because variability between biological replicates can occur for other reasons besides surface inhomogeneity or signal detection artifacts.

### 3.5. Combined Methods

Another approach which combines quantification, loading control normalization and spatial normalization is the “SuperCurve”-based method NormaCurve, published in 2012 by Troncale *et al.* [22]. NormaCurve proposes basically an extended “SuperCurve” model composed of a non-parametric model to quantify relative protein expression from Hu *et al.* [16]. An additive extension takes into account sample effects and spatial effects. Compared to other “Super-Curve”-based models, this one allows a full and reproducible removal of spatial bias as it considers positive control and total protein stained arrays for normalization and spatial covariates for correction of the spatial bias, respectively. The resulting model was further explored to assess the reproducibility of control arrays between slides, the optimal number of replicate spots and the minimally required number of serial dilution steps, as addressed in the following section.

### 3.6. Number of Serial Dilutions Steps

In general, RPPA data analysis relies on serial dilution data of samples to assess the dynamic range of the measurements and to derive valid quantitative data, as outlined by Zhang *et al.* [23]. To assess the optimal number of dilution steps, Troncale and colleagues [22] printed samples as 15-step serial dilutions and used this information as surrogate gold standard. Hence, data of the 2, 3, 5, 6 or 14 upper dilution steps was compared against the surrogate gold standard. Relative expression levels were estimated for each of the resulting dilution curves and compared to the “true” protein concentrations estimated from the two highest number dilution series, comprising 14 and 15 dilutions steps. Dilution series data were compared by cross-validation. A significant improvement of accuracy was observed for dilution series comprising more than three dilution steps. Based on this, authors recommend printing samples as five-step serial dilution series.

### 3.7. Analyte Normalization for Complex Biological Samples

Particularly complex tissue samples such as samples obtained from whole tissue specimens that might include blood vessels or show enrichment with stroma components so that additional normalization measures are required. In that case, analyte normalization, as described by Chiechi *et al.* [27], can correct for sample-to-sample variability but certainly requires suitable controls to permit a valid data analysis.

Most RPPA normalization approaches described here are available as non-commercial software tools (Table 1). In any case, experimentalists and data analysts should make themselves familiar with the requirements of their data analysis pipeline, especially as this also concerns sample preparation, identification of suitable positive and negative controls and the choice of a particular detection method for signal visualization. However, it is also important to notice that data normalization comprising a large number of different steps increases the risk of over-normalization and raises the question of adequate data quality.

### 3.8. Quality Control

Monitoring the quality of raw data constitutes a key element of RPPA data analysis. Variability of data will be observed even under optimized experimental conditions and needs to be addressed with standardized quality control measures. Image quality control checks have mostly relied on the visual examination of slide image files, correlation analysis of technical replicates, inspection of negative control slides detected by omitting the primary antibody and included also quantile-quantile plots comparing negative control slides and actual RPPA slides.

**Figure 3:**
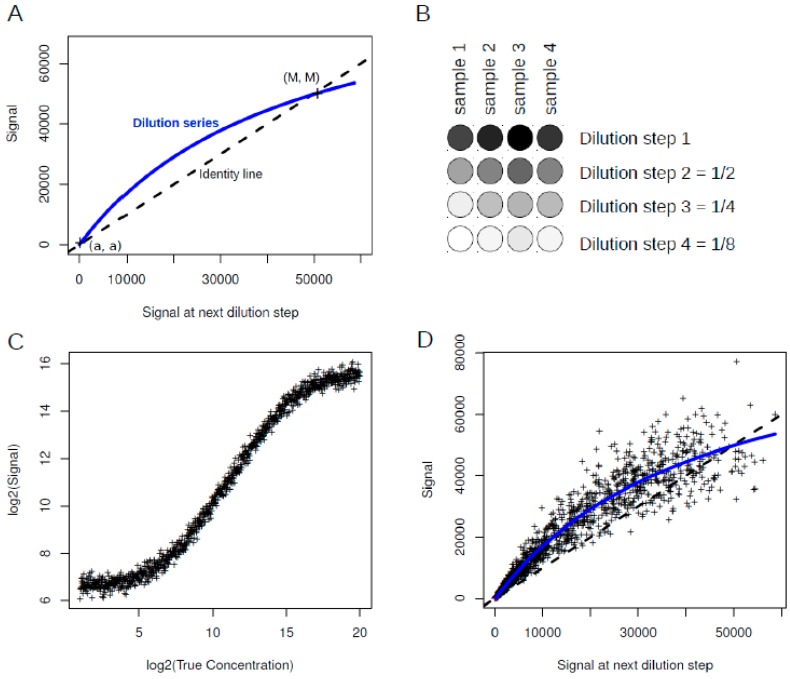
Serial dilution curve. (**A**) In the serial dilution plot the observed signal is plotted against the observed signal at the next dilution step. Dilution series which are very close or identical with the identity line indicate quality problems, as the dilution series fails to generate lowered signals. a and M are the intersection points at background level and saturation level, respectively; (**B**) Example of dilution curves from four samples with different initial concentrations. The dilution steps remain constant; (**C**) Simulated RPPA data generated with the Sips model as presented in Zhang *et al.* [23]; (**D**) Serial dilution curve for simulated data shown in (**C**). The continuous (blue) line corresponds to the serial dilution curve. The dashed (black) line represents the identity line. Scripts for plot generation were taken from [23].

A considerable disadvantage of the visual inspection is a high degree of examiner variability that might produce inconsistent results. Ju and coauthors tackled this problem by setting up an automated data analysis pipeline [20]. Inspection of RPPA images relies on a generalized linear model as a logit to a logistic function returning a likelihood factor that represents slide quality. Evaluation of this automated approach showed prediction accuracies of 84%–87% when compared to a combined evaluation of three RPPA experts, considering the fact of missing a gold standard of RPPA quality control. This approach has been implemented in the “SuperCurve” R package and is available for public use. Since the classifier is array design-specific, it is of limited use in other experimental settings. However, RPPA Core Facilities where experimental settings remain unchanged over many years benefit from such a classifier. The lack of flexibility of this type of approaches portrays the need for standards for RPPA-based targeted proteomics. Individual projects with a platform-tailored experimental design still require running quality control steps manually. In such cases meaningful and intuitive display of raw data results are of great help to complement the visual inspection which might take into account spot shape and size, spot intensity, background intensity, uneven patches as well as variation in the positive controls. Zhang *et al.* [23] suggested to use the “serial dilution curve” as intuitive means for QC (Quality check) as depicted in Figure 3.

The visual inspection of RPPA images and of quantile-quantile plots is the current practice for quality control although it presents a time-consuming procedure and might entail inconsistent results. Consequently, there is definitely a need for automated and flexible quality check approaches for RPPA to exclude low quality images from RPPA data sets.

### 3.9. Further Considerations

Although more than 1000 samples may be printed on a single slide and up to a few hundred different proteins can be probed by RPPA [6,15], certain experimental settings might still involve larger numbers of samples to be analyzed in a high-throughput-fashion. Thus, several slides might be required to accommodate all samples of a study. In addition, in clinical settings it might be of interest to compare results from different studies or across different labs.

In this instance, issues such as inter-slide variability and normalization are pivotal but finding an appropriate normalization approach presents still a challenge [17,36]. Data of positive control spots, serial dilutions and of slides stained for total protein are required for several reasons. This data increases the accuracy of the estimated protein expression levels, adjusts for slide-to-slide variability and compensates for differences in sample loading. Additionally, effects caused by spatial artifacts can be compensated as these might be additive in comparisons of slides from different print runs or from independent signal detection runs. Thus, the experimental design should include a well-defined set of controls shared by RPPA printing runs over many years and by different groups.

Again, the advantage of standardized array design and analysis approaches comes forward and points towards the fact that uniquely designed experimental set-ups might be confounded by a lack of standardization. For this reason, existing methods need to be compared to define an appropriate work flow as standard for RPPA experimentation. Nevertheless, a certain flexibility regarding the experimental design of new projects and new biological questions will require different set-ups. Consequently, flexible methods are needed which in addition can be used in a standardized pipeline for data analysis. Non-flexible tools only developed for in-house solutions do not help to reach this goal in the near future.

## 4. RPPA Data Analysis Tools

Several different software packages have been used for RPPA imaging and data analysis. Most packages were initially developed for microarray data analysis in general. Commercial software packages include Array Pro (MediaCybernetics, Rockville, MD, USA), GenePix Pro (Molecular Devices, Sunnyvale, CA, USA) and Mapix (Innopsys, Carbonne, France). In the last years, it was realized that comprehensive software tools specifically designed for RPPA applications provide more accuracy and improve the user-friendliness for the growing RPPA community. For this reason, exclusively software tools for RPPA data analysis tools were discussed.

Table 1 gives an overview of currently available non-commercial tools and lists the methods implemented in the different approaches (see previous section).

### 4.1. Data Handling

An important, but often underestimated, task of RPPA data analysis is sample management and sample tracking. Most existing solutions of RPPA data analysis tools neglect this aspect. The “SuperCurve” suite provides a tcl/tk-based graphical user interface, which eases the data management for experimental biologists. The other tools expect either basic knowledge with R, are not sufficiently documented or were only implemented as post-analysis tools. Only MIRACLE (Table 1) enables a biologist-friendly web-interface that is easily accessible and supports R with a direct import/export interface for data analysts with experience in programming [30].

The complexity of RPPA data also suggests implementing a data management system for standardization of RPPA data. Such a concept was introduced by Stanislaus *et al.* [39] who tried to form an RPPA Information Management System in 2008, including a reverse phase protein array markup language (RPPAML) to describe, document and disseminate RPPA data. Currently, the project is apparently not further maintained although it would be beneficial for the growing RPPA community. Reasons for this could be the implementation of RPPAML for the commercial software MATLAB while not providing an interface to R methods that are widely used in this field. MIRACLE [30] can currently substitute some of the data handling and sample tracking functionalities needed as it balances user-friendliness and flexibility. However, standardized RPPA data analysis programs are required to handle the complex information associated with proteomic experimentation and to make this information available for public proteomic databases.

### 4.2. Data Integration

Introduction of a commonly accepted RPPA data standard would also be of high importance in regard to studies dealing with cross-platform data analysis. As the awareness for complex biological regulatory mechanisms grows, there is an increased request for “omics” data integration [40]. Investigation of the interplay between different functional cellular levels and of cellular communication can lead to more complete models of physiological and disease states. This rather holistic systems biology approach can thus lead to more specific and personalized treatment options [41].

However, in order to stimulate integrative and translational research, clinical data sets need to be publicly available and processed with standardized pipelines. In addition, communication of integrative study results and integration with clinical decision-making has to be adapted to the idea of personalized treatments. Nevertheless, cross-platform data integration constitutes a challenging, but highly promising and conclusive concept to advance clinical treatment decisions.

## 5. Current State and Future Perspectives of RPPA

Of major value for RPPA experimentation is a thorough knowledge regarding protein signaling networks and antibody specificity as this is the key for successful RPPA experimentation. Unfortunately, the majority of antibody vendors leaves antibody validation to customers and provides limited information about suitable controls. Characterizing and handling large numbers of antibodies therefore constitutes a huge workload for RPPA labs, especially since re-testing has to be carried out with the purchase of each new antibody lot. A major concern is that antibody providers might not be able to maintain their quality in the long run as this might be compromised for the sake of improved financial return.

Different printing and signal detection approaches as well as data analysis tools have evolved since the introduction of RPPA as a highly sensitive large-scale platform for targeted proteomics [15]. The RPPA field would definitely benefit from a comparison of different RPPA platforms to evaluate that the different strategies taken for RPPA return the same results. Such efforts are currently ongoing and its publication will be of value for the field. The question of whether scientific findings such as biomarker signatures can be reproduced on different RPPA platforms or even on other technical platforms, e.g., by mass spectrometry, presents also a highly interesting question. Reviewing the current literature returned encouraging examples.

The analysis of hormone receptor-positive breast cancer employed RPPA to compare proteomic signatures of low- and high-grade breast cancer. The identified biomarker signature comprised caveolin-1, NDKA, RPS6, and Ki-67 and could determine the recurrence risk in patients with ER+ breast cancer implying that it could potentially also be applied to predict a need for chemotherapy [7,42]. Moreover, the clinically relevant and biologically insightful observation that caveolin-1 expression of tumor-associated fibroblasts inversely correlates with clinical outcomes in patients with ER+ breast cancer was already made years ago by using IHC [43]. The observation that also stroma proteins might be identified as part of a biomarker signature supports the idea of understanding cancer as a “tumor organ” with stroma cells as integral partners of the tumor that sustain tumor growth. The prognostic relevance of tumor stroma proteins with respect to grading and patient outcome was also identified by a LC-MS/MS-based analysis of breast cancer specimens. Likewise, the mass spectrometry approach identified different sets of proteins and showed that structural proteins of the tumor stroma are highly abundant in low-grade tumors and additionally identified proteins directly or indirectly associated with transforming growth factor β (TGFβ1) signaling as upregulated in the tumor stroma [44].

RPPA-based profiling of breast cancer specimens including all known histological subtypes, including also HER2+ breast cancer and triple negative breast cancer (TNBC) in addition to ER+ breast cancer samples, identified a small subset of breast cancer patients with relatively high levels of pHER2(Y1248) but without overexpression of HER2. This therapeutically relevant finding showed co-expression of HER2 along with either EGFR or HER3, and hints towards a subgroup of breast cancer patients that can potentially benefit from therapies targeting the full EGFR/ERBB module [45].

Hennessy *et al.* [3] reported six independent groups with distinct proteomic profiles and characteristic overall survival after analyzing 128 breast tumors using 82 antibodies recognizing cancer-relevant proteins and phosphoproteins. Two subgroups showed high level expression of stroma proteins such as collagen VI and caveolin-1, of which collagen VI was also part of a proteomic signature to separate luminal A *vs.* luminal B tumors. The prognostic relevance of stroma proteins emerged also in the analysis of the TCGA work that reported fibronectin, collagen VI, and caveolin-1 as part of stroma signatures named reactive I and reactive II [46,47]. One of the stroma subclusters comprised exclusively luminal A tumors, the second group consisted of luminal A and luminal B tumor, relying on the transcript-based PAM50 score as reference point. Reactive I and reactive II groups did not differ with respect to tumor cell content, and a supervised analysis did not indicate differences with respect to genetic aberrations, e.g., mutation, DNA copy number or methylation.

These findings illustrate the potential of RPPA for biomarker discovery and place it next to established approaches such as IHC and mass spectrometry [48,49]. A direct comparison of RPPA in terms of assay sensitivity with other targeted immunoassay approaches such as the more recently introduced ABCD-technique [50] might be of interest for the field of immunoassays. In this sense, the high sample capacity and high accuracy of RPPA make this platform promising for the validation and quantification of target proteins identified by mass spectrometry, for example. Ideally, these approaches work hand in hand by exploiting the potential of mass spectrometry for *de novo* discoveries and the capacity of IHC to identify the subcellular localization of the protein of interest. Moreover, RPPA appears to be extremely valuable to advance personalized medicine where information on the abundance of targetable receptors, for example, is required. Numerous drugs target receptor tyrosine kinases to date, for example, and the majority of these drug targets can be quantified in clinical tissues by RPPA. Thus, using RPPA for tumor profiling seems to be highly promising also for cancer types not as well characterized as breast cancer. This will open up new therapeutic strategies for patients suffering from rare malignancies or metastatic disease who then may soon have new opportunities to benefit from targeted therapies.
